# Post-stroke care after medical rehabilitation in Germany: a systematic literature review of the current provision of stroke patients

**DOI:** 10.1186/s12913-018-3235-2

**Published:** 2018-06-19

**Authors:** Isabelle Hempler, Kathrin Woitha, Ulrike Thielhorn, Erik Farin

**Affiliations:** 1 Section of Health Care Research and Rehabilitation Research, Medical Center – University of Freiburg, Faculty of Medicine, University of Freiburg, Freiburg, Germany; 2 Catholic University of Applied Sciences Freiburg, Freiburg, Germany

**Keywords:** Neurological rehabilitation, Stroke, Follow-up care, Early supported discharge, Post rehabilitation support, Germany

## Abstract

**Background:**

Although Germany’s acute care for stroke patients already has a good reputation, continuous follow-up care is still not widely available, a problem originating in the strict separation of inpatient and outpatient care. This gap in the German health care system does not just lead to patients’ potential readmission to inpatient care and compromise the sustainability of what they have accomplished during medical rehabilitation; it also places a burden on caregivers.

**Methods:**

To illustrate the current procedures on follow-up care of stroke patients in Germany, a systematic literature search was conducted to gather all available evidence. Research articles in the English or German language were searched between 2007 and 2017. Different study designs ranging from non-experimental descriptive studies, expert reports and opinions were included and categorised by two independent researchers. Relevant data was electronically searched through international and national databases and incorporated in a summary grid to investigate research outcomes and realise a narrative synthesis.

**Results:**

A literature search was conducted to identify all relevant information on how current follow-up care is carried out and evaluated in Germany. We identified no systematic reviews on this topic, but included a total of 18 publications of various original studies, reviews and expert opinions. Included study populations also differed in either: experts, caregivers or stroke patients, including their viewpoints on the outpatient care situation of stroke patients; to capture their need for assistance or to investigate caregivers need and use for assistance. So far there is no standardised follow-up care in Germany, but this review reveals that multidisciplinary cooperation within occupational groups in outpatient rehabilitation is a key item that can influence and improve the follow-up care of stroke patients.

**Conclusion:**

This review was conducted to provide a broadly based overview of the current follow-up care of stroke patients in Germany. Both the new implementation of a standardised, discharge service that supports early support, to be initiated this year and numerous approaches are promising steps into the right direction to close the follow-up gap in German health care provision.

**Electronic supplementary material:**

The online version of this article (10.1186/s12913-018-3235-2) contains supplementary material, which is available to authorized users.

## Background

Experiencing and surviving a stroke is a dramatic and life-changing event for the patient, family members, and caregivers. In Germany, these first-ever stroke events affect approximately 196.000 people per year [1]. Stroke has a mortality of about 63.000 deaths yearly, making it the third most frequent cause of death in Germany [1]. However, due to a good supply of acute health care, death from stroke dropped by about 40% between the years of 1998 to 2008 [2]. This reduction in lost years of life is largely due to the widespread establishment of stroke units.

German stroke units (SU) began to be set up during the last twenty years with the aim of improving emergency care regionally and nationally [2]. Compared to Scandinavian countries and Great Britain, SU in Germany tend to focus more on monitoring the patient during an instable acute phase (e.g. diagnostic and medical treatment), whereas in those countries, the main focus is on a rehabilitative phase [1]. This will soon be the focus in Germany’s acute care. These SU, which are known as comprehensive Stroke Units (cSU), are already up and running in a few hospitals in Germany. Hence, the original SU will then incorporate monitoring during the acute phase and co-ordinate the patient’s mobilisation and early rehabilitation [2]. Whereas Germanys’ acute care for stroke patients is already excellent; the country’s follow-up care is quite inadequate [2]. This has come about because inpatient and outpatient care are strictly separated, making it difficult for stroke patients to organise immediate follow-up care [2].

The German system of rehabilitation is divided into three types [3]: a) medical rehabilitation, b) occupational rehabilitation and c) social rehabilitation. The goal of each is to a) support and assist patients to restore physical and/or psychological functions; b) reintegrate patients who were gainfully employed but unable to return to their previous occupation and c) reintegrate patients in their community. All these types of rehabilitation can take place in different settings, whereas medical rehabilitation usually takes place in the hospital or an inpatient rehabilitation facility. Furthermore, medical rehabilitation in an outpatient setting is also possible, as is occupational and social rehabilitation.

Starting in 1994, different phases of rehabilitation (A to F) were incorporated in Germany’s supplying network of neurological rehabilitation in order to categorise the severity of the patient’s stroke and the subsequent rehabilitation goal [4]. It starts with Phase A, representing the acute phase, followed by Phases B to D, which cover different phases of rehabilitation and aim to promote the patient’s independence. In Phase E, patients have already completed the medical rehabilitation phase, but might still need further treatment in an outpatient setting [4, 5] (Table 1).

**Table 1:** Phases of rehabilitation in Germany

Phase A	Phase B	Phase C	Phase D	Phase E	Phase F
*Acute care*	*Post-acute period – Early rehabilitation phase (inpatient setting)*	*Post-acute period - Rehabilitation phase (inpatient setting)*	*Post-acute period – Rehabilitation and Follow-up treatment care (inpatient or outpatient setting)*	*Occupational rehabilitation and follow-up care*	*Long-term care*
Patients are either on a stroke or an intensive care unit.	Patients still need intensive care but are able to start with complex early rehabilitation measures.	Patients can already actively participate during therapy, but are still in need of high medical and nursing care.	Patients have completed the early mobilisation phase and are mostly independent.	Patients have completed the medical rehabilitation, but need further treatment services in order to be integrated in e.g. working and community life.	Patients need permanent and supportive long-term care.

The above-mentioned phases both categorise each patient’s severity and goals and determine which insurance company is responsible for covering the costs [6].

Objective of this review is to provide the reader with a general overview of how stroke follow-up care is presently provided in Germany, once the patient has finished the medical rehabilitation. Up till now no follow-up programme for stroke patients and caregivers has yet been established as a standard routine in the German healthcare system [7].

### Review question

This literature review was conducted to address the following research question: How is follow-up care of stroke patients currently carried out and evaluated once the patient has completed medical rehabilitation in Germany? Different treatment approaches and evaluations of diverse participants on the topic of follow-up care in Germany need to be identified and summarised. Until now, no systematic review has been conducted. Hence, these findings will be important to address a seldom-discussed but crucial health service research topic in the area of post-stroke care.

## Methods

### Search strategy and eligibility criteria

To obtain a general overview of this health care issue, all studies included in this review were systematically and electronically searched through international and national databases and extracted during the time from September 2016 to January 2017. A search was conducted to identify relevant articles published within the last 10 years (2007–2017) in the English or German language. All study designs and survey methods were included, if the target population was clearly stated as stroke (all stroke types included) patients and covering follow-up stroke care. Articles that evaluated the outcome of different therapeutic interventions, treatment effects or medical treatments during follow-up care were excluded, as it was this review’s objective to investigate general descriptions and evaluations of the provision of follow-up care (see Additional file 1, Prisma checklist). The main search terms are presented in Table 2.

**Table 2:** Sample search terms and search strategy

Stroke	Rehabilitation	Follow-up care	Germany
Key words	Key words	Key words	Key words
[1] cerebrovascular disorders/ or exp. basal ganglia cerebrovascular disease/ or exp. brain ischemia/ or exp. carotid artery diseases/ or exp. intracranial arterial diseases/ or exp. intracranial embolism and thrombosis/ or exp. intracranial hemorrhages/ or stroke/ or exp. brain infarction/ or exp. vertebral artery dissection/ [2] (stroke or cerebrovasc$ or brain vasc$ or cerebral vasc$ or cva$ or apoplex$).tw. [3] ((brain$ or cerebr$ or cerebell$ or vertebrobasilar or hemispher$ or intracran$ or intracerebral or infratentorial or supratentorial or MCA or anterior circulation or posterior circulation or basal ganglia) adj5 (isch?emi$ or infarct$ or thrombo$ or emboli$)).tw. [4] ((brain$ or cerebr$ or cerebell$ or intracerebral or intracran$ or parenchymal or intraventricular or infratentorial or supratentorial or basal gangli$) adj5 (haemorrhage$ or hemorrhage$ or haematoma$ or hematoma$ or bleed$)).tw	[1] rehabilitation* [2] exp. Rehabilitation/ [3] exp. Rehabilitation Centers/ [4] rehabilitat*.ab,ti. [5] exp. Delivery of Health Care/ [6] exp. neurological rehabilitation/	[1] exp. follow up care or aftercare or post stroke care or post rehabilitation support or aftertreatment [2] exp. early supported discharge or post discharge* or exp. outpatient aftercare or Patient Discharge/ or Progressive Patient Care/ [3] home care services/ or home care services, hospital-based/ or home nursing/ [4] (early supported discharge or ESD).tw. [5] ((early or earlier or prompt or accelerate$ or acute or subacute or supported) adj5 discharg$).tw. [6] ((organi?ed. or multidisciplinary) adj5 discharge adj5 team$).tw. [7] ((early or earlier or prompt or accelerate$ or supported) adj5 return$ adj2 home$).tw [8] (hospital$ adj3 home$).tw. [9] hospital rehabilitation unit$.tw. [10] (rehabilitation adj3 home$).tw. [11] (intensive adj2 home adj5 (rehabilitation or support$)).tw. [12] (mobile adj2 team$).tw. [13] organi?ed. home care.tw. [14] ((extended stroke unit adj3 (service$ or care)) or ESUS). tw. [15] ((post-discharge or home rehabilitation) adj5 (support$ or care)).tw. [16] ((early or earlier or acute or subacute or post-discharge) adj5 (community or domiciliary or primary care or home or home-based) adj5 (rehabilitation or support$ or care)).tw.	[1] Germany [2] german* [3] deutsch* [4] deutsch[tt]

Alterations in truncations/ wildcards were possible according to the databases

Our search strategy included search terms related to the diagnosis of stroke, rehabilitation, and follow-up care in Germany. These terms were then combined with the Boolean Operator “AND” to narrow down the search. The databases screened were Medline, CINAHL (viaEBSco), Google Scholar, the Cochrane Library, and two German academic publishing companies (Thieme and the SpringerLink) with many publications in the rehabilitation field. The search strategy was modified to each database’s characteristics. Furthermore, the reference lists of relevant articles were manually revised (snowballing). Studies included in this literature review described problems associated with or evaluated current follow-up care in Germany. Moreover, several studies identified the success of various follow-up care aspects from the perspective of different clinical experts.

### Data extraction and analysis

Data were extracted in a summary grid format by the main researcher (IH). Relevant information comprised general information, research method and research outcome. Additionally two reviewers (IH, KW) independently categorised all selected articles according to the Classification schemes of Shekelle et al. [8] used in the practice guidelines by the National Guideline Clearinghouse (see Table 3). This grading system was chosen as it is a good tool to assess “all available evidence” and categorise a multitude of different study types including expert views and experiences.

**Table 3:** Level of Evidence according to the Classification schemes

Level of Evidence:	
Ia	Evidence for meta-analysis of randomized controlled trials
Ib	Evidence from at least one randomized controlled trial
IIa	Evidence from at least one controlled study without randomization
IIb	Evidence from at least one other type of quasi-experimental study
III	Evidence from non-experimental descriptive studies, such as comparative studies, correlation studies, and case-control studies
IV	Evidence from expert committee reports or opinions or clinical experience of respected authorities, or both

## Results

**Fig. 1:**
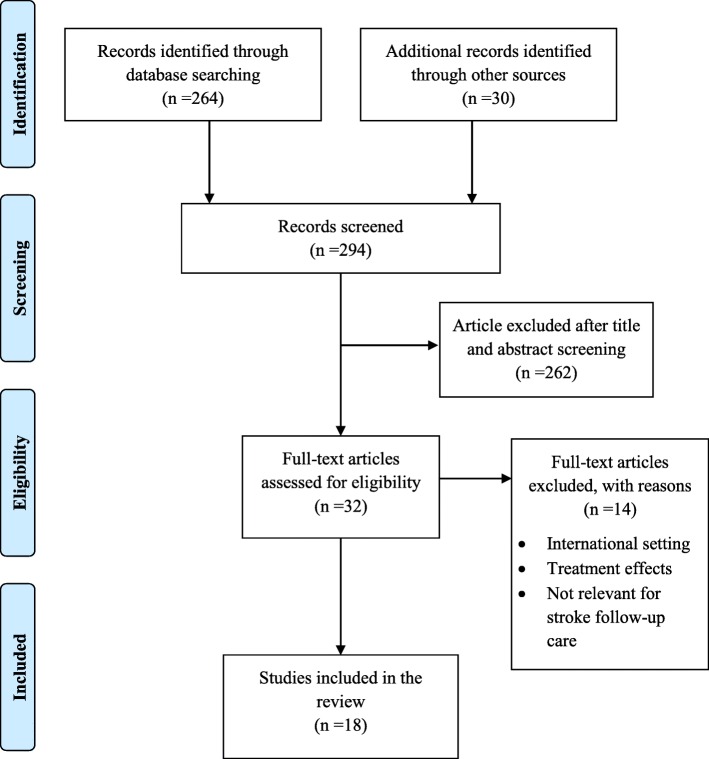
Process and results of the literature search

Our literature search yielded a total of 294 articles addressing stroke follow-up care in Germany. Although no systematic reviews were identified, we selected many empirical studies and overviews written by experts in neurological rehabilitation. After screening and assessing eligibility, we identified 32 articles, 18 of which (17 in German and one in English) were considered and assimilated into this review as they fulfilled our inclusion criteria (Fig. 1). After the categorisation process, most (*n* = 12) of the aforementioned articles were assigned a Grade III, and six articles a Grade IV Level of Evidence.

We had to exclude 14 articles as they dealt with therapeutic treatment effects or the setting was not in Germany. Those we included were extracted from twelve different journals (*n* = 11 national, *n* = 1 international) and varied from quantitative studies (*n* = 7), qualitative studies (*n* = 4) and mixed method (*n* = 1) to expert overviews and policy papers (*n* = 6). After retrieving information from all 18 articles, the following main themes originated: Description of current follow-up care and how it operates and Evaluation of current follow-up care from different views. Our evaluation was also subdivided into themes that reflected different views from clinical experts and caregivers. Major topics were the economic costs of stroke care, positive treatment approaches to enhance follow-up health care provision, and lastly, prospective changes in the law regarding discharge service incorporating early support (Table 4).

**Table 4:** Summary of all publications included in this review

No.	Author & year of publication	Characteristic	Population	Objective	Level of evidence
1	Barlinn et al. 2016	Prospective Pilot study	Patients who experienced a haemorrhagic or ischemic stroke	To investigate the feasibility of a standardised treatment programme for stroke follow-up care	IIa
2	Barzel et al. 2007	Exploratory study	Physiotherapists, occupational therapists	To analyse the outpatient care situation of chronic stroke patients	III
3	Barzel et al. 2008	Exploratory study	General practitioners	To analyse the outpatient care situation of chronic stroke patients	III
4	Düchs et al. 2012	Longitudinal study	Stroke patients who experienced a haemorrhagic or ischaemic stroke, a subarachnoid bleeding or cerebral sinus venous thrombosis and who have been discharged from inpatient rehabilitation	To explore the provision and predictor for therapy during outpatient care	III
5	Heuschmann et al. 2010	Review	First-ever and recurrent stroke patients	To summarise epidemiologic data regarding frequency and care of stroke patients in Germany	IV
6	Hoeß et al. 2007	Prospective longitudinal study	Stroke patients who have been discharged from inpatient rehabilitation	To explore the provision of therapy and technical aids during outpatient care	III
7	Jungbauer et al. 2008	A qualitative longitudinal study	Spouses of stroke patients	To investigate caregivers need for assistance	III
8	Korzilius and Osterloh 2017	Policy paper	–	To improve the early supported discharge management in hospitals	IV
9	Nolte et al. 2008	Longitudinal study	Acute stroke patients	To capture the care situation of patients 4 years after they have suffered a first-ever ischaemic stroke	III
10	Padberg et al. 2016	Prospective Observational study	Stroke patients, caregivers of stroke patients or health professionals	To explore social service requests	III
11	Peschke et al. 2012	Analysis of Health Insurance Routine Data	Stroke patients	To explore the quantity and continuity of physiotherapy and occupational care after inpatient discharge	III
12	Reichert et al. 2017	Review with incorporated expert opinions and focus groups	Data of post-acute stroke patients	To investigate the economic potential of a health care management approach	IV
13	Reuther and Wallesch 2015	Expert view	Stroke patients	To describe participation after stroke	IV
14	Ritter et al. 2012	Review	Stroke patients	To reveal the future perspectives of stroke care	IV
15	Schlote, et al. 2007	Longitudinal study	Stroke patients and caregivers	To investigate caregivers’ acquaintance with, need and use of assistance	III
16	Schlote and Richter 2008	Review	Relatives of stroke patients	To describe the role played by and its associated consequences for relatives of stroke patients	IV
17	Staudacher et al. 2015	Longitudinal study	Stroke patients	To facilitate structured follow-up care for stroke patients	III
18	Sterl and Boehme 2016	Qualitative study	Stroke patients	To evaluate a case management programme and detect measures that positively influence the process	III

### Description of current follow-up care and how it operates

The follow-up care of stroke patients in Germany is primarily carried out through a therapeutic treatment plan [9] that can be prescribed by the general practitioner or a medical specialist, i.e. a neurologist. The amount and type of treatment (provided through a single prescription) always depend on the patient’s diagnosis and condition. Furthermore the patient’s evaluation (as done by the practitioner) plays an important role in determining how much and what sort of treatment is prescribed. These provided treatment prescriptions are offered on a low-threshold service and are easy accessible, nevertheless the demand for descriptions tend to decline in frequency over time [9]. According to Düchs et al., the main treatment prescribed is physiotherapy, followed by a combination of physiotherapy and occupational therapy [10]. However, occupational therapy on its own is not prescribed as often as physiotherapy [9]. Reuther and Wallesch have the opinion that having access to these prescriptions and continuing outpatient therapy after inpatient rehabilitation are very important to stabilise the improvement achieved during rehabilitation [11]. Düchs et al. reported that there is evidence that treatment combinations improve follow-up care [10]. However, Hoeß et al. found several influencing factors contributing to the repeated provision of treatment prescriptions, for instance the patient’s younger age; more frequent visits to the physician or having the specific treatment goal to improve mobility [12]. The study by Nolte et al. shows that general practitioners are usually the stroke patient’s principal point of contact [13] once inpatient rehabilitation has been completed. Multidisciplinary cooperation between specialists or occupational groups is not the rule during the outpatient care of stroke patients [13]. Only half of the treatment recommendations from clinical physicians for outpatient care have actually been carried out a year after patients completed their medical rehabilitation [10] and have returned home. In 2008, Barzel et al. gathered general practitioners’ points of views on the topic of follow-up care and found that the problem in outpatient care originates during inpatient care [14].

#### Stroke’s health-economic burden (follow-up care)

In Germany, the amount of health-care money spent on acute care, rehabilitation, and follow-up for stroke patients is among the highest in the German health care system. According to Düchs et al. [10], these costs can rise up to approximately 43.000 Euro per person. Hence, according to Heuschmann et al. [1], in the upcoming 20 years 108 billion Euros will be spent on the health care of patients suffering a first-ever ischaemic stroke. On the other hand, Reichert et al. [15] found that a care-management approach during follow-up can lower such long-term health care costs over time by about one million Euros.

### Evaluation of current follow-up care from different perspectives

#### Perspectives of clinical experts

This section provides an overview of how clinical experts evaluate the provision of outpatient care of stroke patients, starting out with physio- and occupational therapists and how they perceive the quality of follow-up care. According to Barzel et al., therapists and physicians have identified a critical shortage of outpatient care [16] - namely that the therapeutic success observed during rehabilitation may be at risk. Another point of dissatisfaction reported by outpatient therapists is the lack of multidisciplinary co-operation, e. g. with physicians in private practice [16]. This should be included in outpatient care, as stroke is such a complex disease that patients often need therapy from several different medical disciplines [13]. According to general physicians (GPs), stroke patients are not well prepared for their return back home after completing inpatient rehabilitation [14]. This problem has to do with both poor outpatient care provision and partly inpatient care [14]. The study by Barzel et al. emphasises that stroke patients’ care cannot succeed if carried out by only one occupational group, it rather requires a multidisciplinary approach from different professional groups for the individual patient and his or her specific needs [16].

#### Perspectives of informal caregivers

This section covers the important role, as well as the heavy burden carried by caregivers when it comes to follow-up care. Schlote et al. state that fundamental assistance to and support for stroke patients are usually provided by a spouse or a child [17]. The patient’s disease means major life changes personally, as well as for the responsible caregivers. However, stroke can lead to a change in relationships and alterations in family roles [17]. Such emotional modifications within a family can lead to excessive demands and to a feeling of helplessness in those family members involved in caring for a stroke patient [17]. So far there is no standardised support for caregivers in Germany when it comes to follow-up care, even though many suffer from psychological and physical stress [17]. Caregivers, who are usually the main person providing support [16], are rarely considered as a resource that can provide key information regarding the patient’s social or familial needs [18], which subsequently benefit the discharge process back home. Barzel et al. describe that the burden for caregivers is so intense, that even therapists, who are primarily treating the patient, feel the need to serve caregivers as a contact person for support [16].

Regarding health care costs, the study by Schlote and Richter highlights an important point about economising. Generally speaking, it is the involvement of caregivers that saves the German health care system an enormous amount of money [19]. Their involvement can include the co-ordination and organisation of and possible transportation to follow-up appointments with physicians or therapists. Such responsibilities lead to a strain on caregivers and could influence their social life. Caregivers may even have to give up their occupation in order to take care of the stroke patient, which can lead to a huge reduction in income affecting the whole family [19].

### Different treatment approaches to improve follow-up care

As no follow-up programme for stroke patients and caregivers has yet been established as a standard routine in the German healthcare system [7], several studies evaluated different follow-up approaches to help patients and their caregivers during the transition phase from medical rehabilitation back into their home environment. These projects included counselling by social workers at a Stroke-Service-Point (SSP) [20], an organised stroke nurse [21] and structured help by a case manager [18, 22].

#### Stroke service point

The “Stroke-Service-Point” (SSP) was a point of contact to receive information operated by social workers and situated in Berlin’s centre, or more precisely on a hospital campus. It was accessible for every stroke patient and caregiver, as well as any health care practitioner. Different enquiries regarding medical rehabilitation services, assistance with reintegration back home or back into working life, as well as many other topics could be discussed with the social workers [20]. Padberg et al. reveal that mainly female caregivers made use of this Service-Point to ask for assistance. Topics that were most often addressed were services concerning outpatient care (such as adapting the home) and outpatient rehabilitation. Padberg et al. also reported that patients and their relatives or caregivers are often uninformed about services they are entitled to and about how to establish contact. They also report trouble obtaining these services because of the bureaucracy [20].

#### Stroke nurse

A study by Staudacher et al. [21] shows that individual service offered by a stroke nurse can not only lead to less stroke recurrence and lower consequential costs, it is also positively received by patients and caregivers. They report that the stroke nurse recruited patients while they were being hospitalised on a stroke unit. Once the patients agreed to participate in their study, the nurse initiated follow-up care procedure such as scheduling follow-up appointments with physicians, secondary prophylactic measures, and helping with psychosocial problems [21]. This study shows that although the stroke nurse only visited stroke patients twice a year (after 3 and 6 months), this intervention still increased the patients’ follow-up visits with a neurologist and lowered the rate of a recurrent stroke by 5.5% within the first year after their first stroke.

#### Case management

The study by Sterl and Böhme assessed a case management programme [18], indicating good future prospects on how gaps in follow-up care can be filled. This programme is offered by a private insurance company and consists of supervision, support, and arrangements with physicians and therapists, provided by a social worker through phone calls. Within this study, patients reported feeling abandoned, due to the fact that they had been given the information of the discharge process on their actual day of discharge. Additional outcomes of this study demonstrate that caregivers do still not get involved in the discharge process, even though they are a useful and important resource when it comes to keeping abreast of the patient’s family and social situation. Patients also reported that they were unable to continue with therapy 2 to 4 weeks after they have returned home because they had to find an outpatient therapist and make appointments themselves.

### Early supported discharge service

Each inpatient rehabilitation facility is currently responsible for organising its own early supported discharge service. However, the Federal Joint Committee (one of the “highest decision-making bodies of self-government” of physicians and hospitals) [23] recently passed a new law calling for standardised discharge management that will be mandatory for all the rehabilitation clinics and experts involved.

This standardised approach includes a discharge assessment carried out by clinical physicians to identify patients’ follow-up needs before discharge to issue prescriptions for medications; certain aids; to declare the patient’s temporary work-disability, or to order follow-up therapy [24]. For patients needing more individual care, follow-up appointments with the associated general practitioner or specialist will also be scheduled by the clinic [24]. To consult the practitioner in the clinic, his or her contact number is included on the discharge report. Although this new service has been much criticised for being overly bureaucratic, it will soon be implemented and help closing the gap between in- and outpatient rehabilitation.

## Discussion

A literature search was conducted to identify all relevant information on how current follow-up care is carried out and evaluated in Germany. It revealed that multidisciplinary cooperation within occupational groups in outpatient rehabilitation, the role of caregivers, and the aforementioned follow-up approaches (Stroke service point, Stroke nurse, and Case management) are the key items that can influence and improve the follow-up care of stroke patients.

The most recent evidence from various clinical experts shows that stroke patients are still not being cared for well enough once they have returned home. This problem’s origin tends to lie in the inpatient rehabilitation context, and it persists long after patients become outpatients. This problem may develop because patients are not well prepared by early supported discharge programmes, which all neurological rehabilitation facilities should offer. Moreover, this review reveals that good follow-up care depends on both the rehabilitation facility and on multidisciplinary cooperation between the facility, caregivers and therapists. To ensure good, consistent cooperation, time should be made for routine case reviews on a daily basis to discuss the patient’s current state of health and needs where required. Additionally this time for discussion should also be reimbursed.

The approaches mentioned above (e.g., the stroke nurse or case management) highlight an important first step towards better support for stroke patients and caregivers throughout the process of discharge and reintegration back home. The outcomes reflect a lower rate of stroke recurrence, as well as positive feedback from patients thanks to frequent supervision and support offered by a responsible stroke nurse. This shows that the regular supervision and support by a responsible person both lowers the risk of suffering another stroke and makes patients feel noticed, accepted, and looked after, all factors that can benefit their health. Additionally the results also reveal that patients desire a contact person who is responsible and approachable for them throughout the transition phase from being an inpatient to outpatient. Establishing a responsible contact person has both positive health benefits and is welcomed by patients.

Another important aspect is the caregiver’s role, as they are usually the main provider of support. Mainly relatives, and primarily women, are closely involved in getting advice on different services regarding follow-up care [20]. Even though most caregivers desire active involvement in follow-up care, they are unfortunately still not regarded as a resource. Therefore it is very important to involve and integrate caregivers as early as possible in the discharge process. Integrating caregivers needs to be a crucial step in the early phase of rehabilitation as they are already providing care with no guidance from stroke experts; a situation associated with a higher risk for them to become physically or emotionally distressed. Hence, this problem should be avoided by integrating them early in the process. Additionally they should also be reimbursed for their efforts, as they receive too little financial support, potentially leading to financial insecurity. Schlote and Richter [19] published a comprehensive review about the burden of caregivers that accompanies caring for a spouse, sibling or parent who suffered a stroke. This serious problem concerning relatives who automatically become informal caregivers has already been acknowledged in the Netherlands where, as a result, recommendations for caregivers are now incorporated in national guidelines advising family social workers to offer support through information and advice [19].

This review was conducted to provide a broadly-based overview of the current practice of follow-up care for stroke patients. Based on all the identified evidence, the efficacy of the standardised early supported discharge service should be investigated once it is initiated by the Federal Joint Committee this year. The early supported discharge service will hopefully lead to an easier and quicker means for patients to receive therapy after an inpatient rehabilitation programme.

### Limitations

There are a several limitations associated with this systematic review. First of all, the relevant search strategy was conducted; articles were screened and subsequently assessed by only one author (IH). Thus it is possible that other search terms would have led to different outcomes. As this review incorporated a diverse range of publications, it is difficult to generalise. Furthermore, the evidence and information reported is very heterogeneous, often because of various methodological study approaches. Nevertheless, the chosen studies still report positive research outcomes regarding follow-up care approaches. This review is merely the first step towards a compilation of follow-up approaches regarding stroke care.

## Conclusion

This review offers an overview of the latest follow-up care standards in Germany and shows that closing the health-service gap between the strictly separated in- and outpatient rehabilitation sectors remains a long-term process. Nevertheless, follow-up care needs to be improved by taking different follow-up approaches and encouraging close cooperation and communication between occupational groups in both rehabilitation settings, as well as by integrating responsible caregivers during the early rehabilitation phase. Such integration measures and co-operation should therefore be anchored in the process of the early-support discharge service and during follow-up care.

## Additional file

Additional file 1: PRISMA 2009 Checklist. (DOC 64 kb)

## Abbreviations

cSU: Comprehensive stroke units
SSP: Stroke-service-point
SU: Stroke units
